# Cytological evidence of *BSD2* functioning in both chloroplast division and dimorphic chloroplast formation in maize leaves

**DOI:** 10.1186/s12870-019-2219-7

**Published:** 2020-01-09

**Authors:** Heying Li, Mei Bai, Xingshan Jiang, Rongxin Shen, Huina Wang, Haiyang Wang, Hong Wu

**Affiliations:** State Key Laboratory for Conservation and Utilization of Subtropical Agro-bioresources, South China Agricultural University, Guangzhou, 510642 China

**Keywords:** Maize, C_4_ photosynthesis, Kranz anatomy, *BSD2*, Chloroplast division, Rubisco

## Abstract

**Background:**

Maize *bsd2* (*bundle sheath defective2*) is a classical C_4_ mutant with defective C_4_ photosynthesis, accompanied with reduced accumulation of Rubisco (ribulose bisphosphate carboxylase oxygenase) and aberrant mature chloroplast morphology in the bundle sheath (BS) cells. However, as a hypothetical chloroplast chaperone, the effects of BSD2 on C_4_ chloroplast development have not been fully examined yet, which precludes a full appreciation of *BSD2* function in C_4_ photosynthesis. The aims of our study are to find out the role of*BSD2* in regulating chloroplasts development in maize leaves, and to add new insights into our understanding of C_4_ biology.

**Results:**

We found that at the chloroplast maturation stage, the thylakoid membranes of chloroplasts in the BS and mesophyll (M) cells became significantly looser, and the granaof chloroplasts in the M cells became thinner stacking in the *bsd2* mutant when compared with the wildtype plant. Moreover, at the early chloroplast development stage, the number of dividing chloroplasts and the chloroplast division rate are both reduced in the *bsd2* mutant, compared with wild type. Quantitative reverse transcriptase-PCR analysis revealed that the expression of both thylakoid formation-related genesand chloroplast division-related genes is significantly reduced in the *bsd2* mutants. Further, we showed that BSD2 interacts physically with the large submit of Rubisco (LS) in Bimolecular Fluorescence Complementation assay.

**Conclusions:**

Our combined results suggest that *BSD2* plays an essential role in regulating the division and differentiation of the dimorphic BS and M chloroplasts, and that it acts at a post-transcriptional level to regulate LS stability or assembly of Rubisco.

## Background

On the earth, although only less than 5% of terrestrial plants can use C_4_ photosynthesis, they give a quarter of the primary productivity [1]. C_4_ plants have higher utilization efficiency of water and nitrogen than C_3_ plants, due to a series of biochemical and anatomical modifications occurred in C_4_ plants [2, 3]. A characteristic feature of C_4_ plants is formation of Kranz anatomy, whereby concentric wreaths of mesophyll (M) and bundle sheath (BS) cells surround closely spaced veins in the leaf (Kranz is German for wreath) [4]. The Kranz anatomy provides a structural framework for compartmentalization of photosynthetic enzymes and proteins in two morphologically and physiologically distinct C_4_ cell types, the BS cell and M cell. Particularly, ribulose-1,5-bisphosphate carboxylase/oxygenase (Rubisco), the enzyme that catalyzes the fixation of CO_2_ for photosynthesis, exists exclusively in BS chloroplasts [5–7]. It is believed that compartmentation of Rubisco away from atmospheric CO_2_ confers C_4_ plants a photosynthetic advantage under high light/temperature growth conditions or arid lands [1, 8].

Considering the importance of C_4_ photosynthesis, there is tremendous interest in understanding how the development of Kranz anatomy is regulated, which is essential for the genetic engineering of C_3_ crop species (such as rice) with C_4_ photosynthesis characteristics [9–11]. Maize (*Zea mays*) is a typical NADP-ME-type (NADP-malic enzyme) C_4_ plants with a relatively simple Kranz structure characterized by a single BS layer with centrifugally distributed agranal chloroplasts. The simplicity and the well-developed genetic resources have made maize an attractive model for studying the biology of C_4_ development [12]. The M chloroplasts in maize are starchless and possess numerous grana, whereas the BS chloroplasts accumulate starch grains and their thylakoids are largely unstacked [13, 14]. The initial carbon fixation happens in M chloroplasts, where phosphoenolpyruvate (PEP) is carboxylated by phosphoenolpyruvate carboxylase (PEPCase) to form oxaloacetate (OAA). Then, OAA is metabolized into malate and diffused into the BS cells in order to increase CO_2_ concentration around Rubisco. This particular mechanism enhances the carboxylation reaction of Rubisco, which significantly increased the photosynthetic yield [15, 16].

To identify the regulators of the Kranz anatomy development, mutagenized maize populations were screened for mutations that specifically disrupt the photosynthetic enzyme accumulation patterns in either bundle sheath or mesophyll cells [17]. The *bundle sheath defective2*(*bsd2*) mutant is defective in C_4_ photosynthesis and it has abnormal BS chloroplasts and reduced accumulation of Rubisco [18]. Molecular cloning revealed that *BSD2* encodes a chloroplast-targeted protein which shares homology with the DnaJ class of molecular chaperones [5]. Further studies showed that BSD2 is not involved in general photosynthetic complex assembly or protein import. In the *bsd2* mutants, although the Rubisco proteins could not be detected, the chloroplast-encoded Rubisco large subunit transcript (*rbcL*) was abundant and associated with polysomes in both the M and BS cells. Therefore, it was suggested that BSD2 plays a direct role in the post-transcriptional control of *rbcL* transcripts accumulation and/or translation, and an indirect role in the maintenance of chloroplast structure in the BS cells. On the other hand, it was reported that chloroplast structure in M cells is not perturbed in the *bsd2* mutant [5, 18]. However, the biochemical function of BSD2 and its regulatory mechanism in dimorphic chloroplast development have remained essentially unknown.

In this study, we showed that the BS chloroplasts have more grana, but their number decreased significantly in the *bsd2* mutant, compared with the wild type. In addition, we found that M chloroplasts are also defective in the *bsd2* mutant, characterized with thinner grana. Moreover, we found that at the proplastid stage, the number of dividing plastids is abnormal in the *bsd2* mutants. Together, our results suggest *BSD2* plays an important role in regulating both the division and formation of dimorphic chloroplasts in maize leaves, thus adding new insights into our understanding of C_4_ biology.

## Results

### Mutation of *BSD2* Disturbs chloroplast grana stacking and starch accumulation

**Fig. 1:**
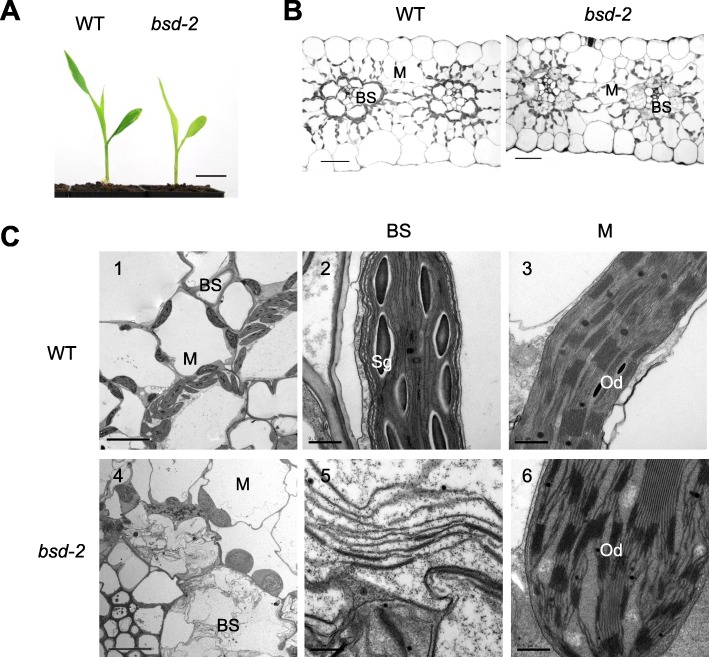
Morphological and anatomical study of 12-day-old wild-type and *bsd2* mutant plants. **a** Morphology of 12-day-old wild type and *bsd-2* mutant plants (scalebar:4cm). **b** Cross sections of the third leaf tip of the wild type and *bsd2* plants. Scalebars:20μm. **c** Ultra structure of the BS and M chloroplasts in the third leaf of the wild type and *bsd2* plants. BS: sheath cells, M: mesophyll cells. Scale bars: 2 μm (1 and 4) and 0.5 μm (2, 3, 5 and 6). Sg, starch granule, Od, osmiophilic droplet.

To investigate the roles of *BSD2* in the regulation of leaf development, we examined the leaf anatomy in twelve-day old *bsd2* mutant and wild type plants (Fig. 1a). Light micrograph observation showed that the M and BS cells of the Kranz anatomy were well developed in leaves of both wild type and the *bsd2* mutant (Fig. 1b). However, the distribution of the chloroplasts in BS cells of wild type and the *bsd2* mutant differed significantly (Fig. 1b). In wild type, most of the BS chloroplasts were located close to the M cells, while BS chloroplasts were randomly distributed in the *bsd2* mutant. On the other hand, M chloroplasts were randomly distributed along the cell wall and no significant difference was found between wild type and the *bsd2* mutant (Fig. 1b).

We further investigated whether the *bsd2* mutation affects the ultra-structure of the mature chloroplasts in the *bsd2* mutant (Fig. 1c). Electron microscopy observation showed that the wild type BS chloroplasts were oval-shaped, with unstacked stromal lamellae and large starch granules of high electronic density (Fig. 1 c1, c2). M chloroplasts in wild type were disk-shaped, but had well developed stacked thylakoids and accumulation of small starch granules (Fig. 1 c1, c3). Compared with the wild type, BS chloroplasts in *bsd2* mutant were swollen or damaged, and the thylakoid system was loosely arranged (Fig. 1 c4, c5). Moreover, no starch granules were found in BS chloroplasts (Fig. 1 c5). In addition, M chloroplasts of *bsd2* were also swollen with the thinnergrana, and the thylakoid system was more loosely arranged when compared with M chloroplasts in wild type (Fig. 1 c4, c6).

### Mutation of *BSD2* disturbs chloroplast development

**Fig. 2:**
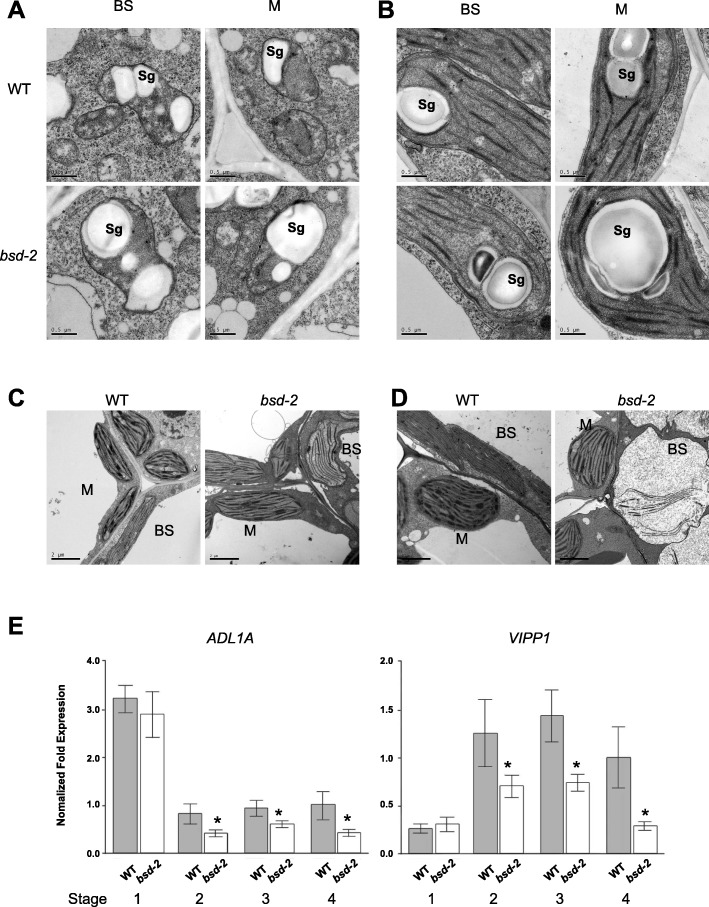
Morphological and anatomical study of chloroplasts in bundle sheath cells (BS) and mesophyll cells (M) of the wild-type and *bsd2* mutant plants. **a** Ultra structure of the chloroplasts (proplastid stage) in the first leaf of 6-day-old wild type and *bsd2* plants. Sg, starch granule. Scale bars: 0.5 μm. **b** Ultra structure of the chloroplasts (grana differentiation stage) in the first leaf of 7-day-old wild type and *bsd2* plants. Scale bars: 0.5 μm. **c** Ultra structure of the chloroplasts (dimorphism formation stage, on the second day after the leaves have grown out of the coleoptile) in the first leaf of 8-day-old wild type and *bsd2* plants. Note reduction in thylakoid stacking in BS compared to Fig. 2b. Scale bars:2 μm. **d** Ultra structure of the chloroplasts (maturation stage, on the third day after the leaves have grown out of the coleoptile) in the first leaf of 9-day-old wild type and *bsd2* plants. Scale bars: 2 μm. **e** Transcript levels of selected thylakoid-biogenesis genes (*ADL1A and VIPP1*) were determined by quantitative RT-PCR in wild-type an dbsd-2 mutant. The error bars represent SD. Stage1: proplastid stage, Stage 2: granal differentiation stage, Stage 3: dimorphism formation stage, on the second day after the leaves have grown out of the coleoptile, Stage4: maturation stage, on the second day after the leaves have grown out of the coleoptile, Stage4: maturation stage, on the third day after the leaves have grown out of the coleoptile. Asterisk means significantly difference that assessed by Student’s t-tests, *p<0.05.

To understand how the defects in BS and M chloroplasts occurred in the *bsd2* mutant, we conducted a comparative developmental time course study of the chloroplast (or plastid) ultrastructure in wildtype and the *bsd2* mutant. Several key developmental stages of chloroplast were examined, including the proplastid stage (leaves still enclosed in the coleoptile, six-day old seedlings), chloroplast-grana differentiation stage (on the first day of leaves growing out of the coleoptile, seven-day old seedlings), the chloroplast dimorphism formation stage (on the second day after the leaves have grown out of the coleoptile, eight-day old seedlings), chloroplast maturation stage (on the third to fifth day after the leaves have grown out of the coleoptile, nine-to-eleven-day old seedlings). At the proplastid stage, proplastids varied in shape and were rich in starch granules and only had weak prothylakoids in both BS and M chloroplasts. No significant difference was found between wildtype and the *bsd2* mutant plants (Fig. 2a). At the chloroplast-grana differentiation stage, both BS and M chloroplasts developed stacked grana thylakoids and unstacked stromal thylakoids in both wildtype and the *bsd2* mutant (Fig. 2b). Still, no significant difference was found in chloroplasts between wildtype and the *bsd2* mutant plants at this stage. At the chloroplast dimorphism formation stage, on the second day after the leaves have grown out of the coleoptile, an increase in grana stacking (thylakoid membrane layers per granal stack) was found in the M chloroplasts in both wildtype and the *bsd2* mutant, while the BS chloroplast had a striking reduction in thylakoid stacking in both wildtype and the *bsd2* mutant. By this stage, chloroplast dimorphism was clearly formed (Fig. 2c). Notably, the thylakoid membranes of BS chloroplasts were more loose in the *bsd2* mutant, when compared with the wild type (Fig. 2c). At the chloroplast maturation stage, the loosening of BS thylakoid membranes became more evident, accompanied by abnormal expansion of BS chloroplasts in the *bsd2* mutant (Fig. 2d). At this stage, the BS chloroplasts in both wild type and the *bsd2* mutant had developed unstacked thylakoid membranes. An increase in grana stacking was found in the M chloroplasts of both wildtype and the *bsd2* mutant. However, the thylakoid membranes were a little bit more loose, and the grana stacking was thinner and wider in the *bsd2* mutant when compared with the wild type (Fig. 2d). These observations suggest that mutation in *BSD2* disturbs development of both BS and M chloroplasts at the maturation stage, and the disturbance was much more severe in the BS chloroplasts.

We next performed quantitative RT-PCR to measure the transcript levels of *ADL1A* (encoding a dynamin-like protein) and *VIPP1* (encoding the vesicle-inducing protein VIPP1), both of which encode structural components of thylakoid during chloroplast development [19, 20]. The transcript levels of both *ADL1A* and *VIPP1* were significantly reduced in *bsd2* relative to the wild type, during the chloroplast-grana differentiation stage, dimorphism formation stage and the maturation stage (Fig. 2e), suggesting that *BSD2* plays a role in promoting the expression of thylakoid formation genes.

### *bsd2* mutant has reduced chloroplast number in BS and M cells

**Fig. 3:**
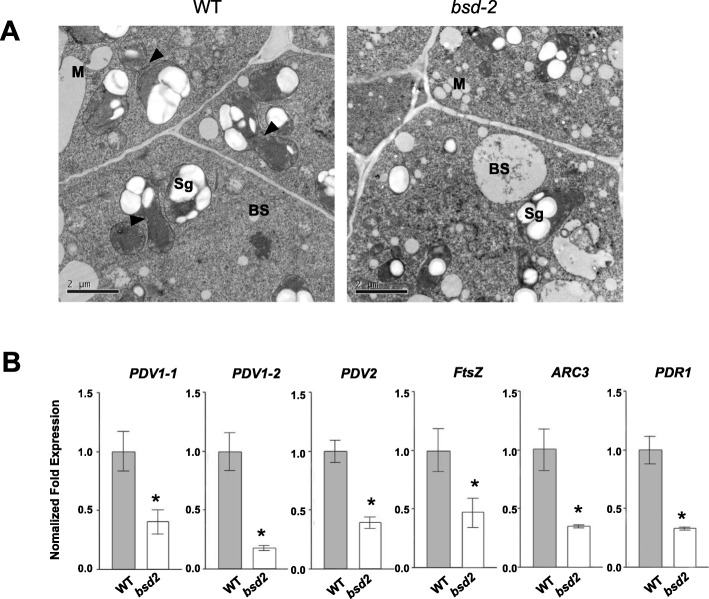
Chloroplast division is affected in the *bsd2* mutant **a** Ultra structure of the chloroplasts (proplastid stage) in the first leaf of 6-day-old wild type and *bsd2* plants. Black triangles indicate dividing chloroplasts. BS, bundle sheath cell; M, mesophyll cell. Scale bars: 2μm. **b** Transcript levels of selected chloroplast-division genes were determined by quantitative RT-PCR in the first leaf of 6-day-old wild type and *bsd2* plants. The error bars represent SD. Asterisk means significantly difference that assessed by Student’s t-tests, *p<0.05.

To test whether chloroplast division is affected inthe *bsd2* mutant, we examined the ultrastructure of the chloroplasts (proplastid stage) in the first leaf of 6-day old wild type and *bsd2* seedlings. Our results showed that wild type plants had more dividing chloroplasts than *bsd2* (Fig. 3a). Next, we compared the chloroplast division rate (measured as the percentage of dumb-bell-shaped chloroplasts per randomly selected chloroplasts in leaf) in *bsd2* and wild type at the proplastid stage. A decrease in chloroplast division rate (by 66.4% for BS chloroplasts and by 40.3% for M chloroplasts, n = ~ 100 chloroplasts from 20 cells of 8 samples) was found in the *bsd2* mutant (Table 1), suggesting that *BSD2* plays a role in promoting chloroplast division at the early developmental stage. We further compared the chloroplast number per cell at the maturation stage, and found a decrease of chloroplasts per cell (25.3% in BS chloroplasts and 18.2% in M chloroplasts, *n* = 20 cells) in the *bsd2* mutant, compared with the wild type (Table 2). These observations indicate that *BSD2* promotes chloroplast division in both M and BS cells.

**Table 1:** Comparison of dividing proplastid number per cell in wild type (WT) and *bsd-2* mutant

		Dividing proplastid number	
		Average ± SD (n = 20)	p(t-test)
BS	BS WT	1.46 ± 0.88	0.001**
	*bsd2*	0.49 ± 0.33	
M	WT	1.09 ± 0.73	0.002**
	*bsd2*	0.65 ± 0.42	

Asterisk means significant difference, **p < 0.01

**Table 2:** Comparison of mature chloroplast number per cell at the maturation stage in wild type (WT) and *bsd-2* mutant

		Chloroplasts number	
		Average ± SD (*n* = 20)	p(t-test)
BS	BS WT	48.6 ± 8.5	0.000**
	*bsd2*	36.3 ± 8.8	
M	WT	29.1 ± 4.5	0.002**
	*bsd2*	23.8 ± 5.6	

Asterisk means significant difference, **p < 0.01

We next performed quantitative RT-PCR to measure the transcript levels of genes encoding structural components of the chloroplast division apparatus [21]. The transcript levels of *PDV1–1* (Plastid Dividing 1–1), *PDV1–2* (Plastid Dividing 1–2), *PDV2* (Plastid Dividing 2), *FtsZ* (Filamenting temperature-sensitiveZ), *ARC3* (Accumulation and Replication of Chloroplasts3) and *PDR1* (Plastid-Dividing Ring 1) were significantly reduced in *bsd2* relative to the wild type plant (Fig. 3b), suggesting that *BSD2* also plays a role in promoting the expression of chloroplast division-related genes.

### BSD2 interacts with Rubisco large subunit (LS)

**Fig. 4:**
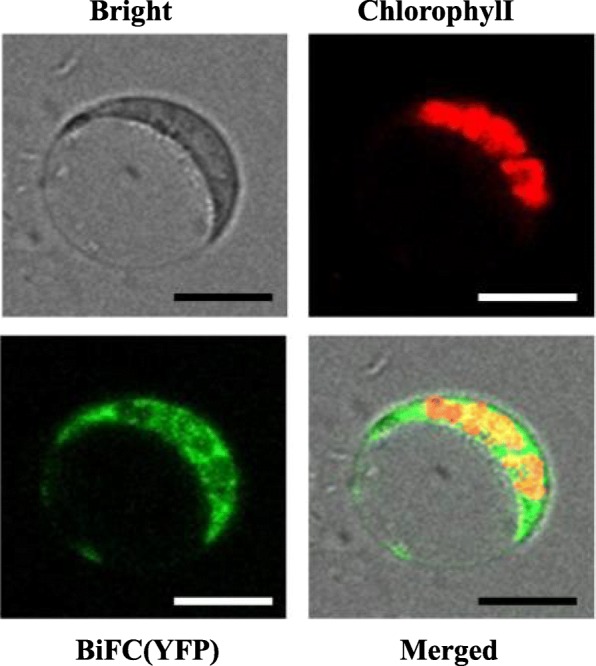
BiFC assay shows that BSD2 interacts with LS in plant cells. Bright, bright light; Chlorophyll, chloroplast chlorophyll auto fluorescence; YFP, YFP fluorescence shows that BSD2 interacting with LS *in vivo*. Scalebar, 10 μm

The Rubisco large subunit (LS) is encoded by the chloroplast *RBCL* gene. BSD2 has been hypothesized to act at a co- or post-translational step during LS synthesis [22–24]. Doron (2014) first indicated an association between BSD2 and the nascent LS peptide in Chlamydomonas based on biochemistry and molecular biology [25]. However, cytological evidence supporting this notion is still lacking. To verify whether BSD2 interacts with LS in vivo, we preform Bimolecular Fluorescence Complementation (BiFC) assay. Our results showed that indeed BSD2 interacted with LS in vivo (Fig. 4). These observations add evidence to support the notion that BSD2 acts at a posttranscriptional level to regulate LS stability or assembly of Rubisco.

## Discussion

### Cytological evidence for *BSD2* functioning in regulating the development of both BS and M chloroplasts

*BSD2* appears to be present only in green algae and plants, suggesting that it has evolved after the endosymbiotic event leading to the evolution of chloroplasts [26]. Previous studies showed that the mutation in *BSD2* leads to abnormal BS chloroplasts (abnormal expansion and with loosen thylakoid membranes inside) and decreased chloroplast coverage in BS cells [18, 27], however, how *BSD2* regulates chloroplast development has remained mysterious. In addition, it was reported that *BSD2* does not affect M chloroplast development [5]. In this study, we found that the mutation in *BSD2* not only affects BS chloroplast development, but also affects M chloroplast development. Our results showed that the BS chloroplasts have more grana, but their number decreased significantly in the *bsd2* mutant, compared with the wild type. In addition, we found that M chloroplasts are also defective in the *bsd2* mutant, characterized with thinner grana. This notion is further supported by the observed decrease in the expression of genes related to thylakoid formation. Furthermore, we found that chloroplast division is also affected in the *bsd2* mutant as we observed fewer dividing proplastids in *bsd2* during the early developmental stage (Fig. 3a) and that the division rate of chloroplasts is decreased in the bsd2 mutant (Table 1). This notion is also supported by the observed reduced expression of several genes related to chloroplast division (Fig. 3b). Thus, our combined results are consistent with the earlier report that chloroplast coverage is reduced in BS cells, likely due to a combinatory effort of decreased chloroplast division rate and abnormal chloroplast development in BS cells. Our findings that *BSD2* also affects the division rate and development of M chloroplast uncover a previously unrecognized role of *BSD2 in* regulating kranz anatomy development. In this regard, it is particularly interesting to note that a recent study showed that cell type specific expression of *BSD2* in BS or M cells driven by the *RBCS* or *PEPC* promoters all could complement Rubisco deficiency and seedling lethality conferred by the *bsd2* mutation [27]. These new findings raise a possibility that *BSD2* mRNA or its encoded product may be partitioned between the BS and M cell types. This will be an interesting topic for future studies.

It has been reported that the M chloroplasts of all C_4_ species are randomly distributed along the cell wall, but BS chloroplasts are located either in a centripetal (close to the vascular tissue) or in a centrifugal (close to M cells) pattern, depending on the species [28]. Interestingly, we found that most of the BS chloroplasts are located close to the M cells in wildtype, however, BS chloroplasts are randomly distributed in the *bsd2* mutant, suggesting that *BSD2* may also play a role in determining the distribution pattern of BS chloroplasts. However, the detailed mechanism remains to be explored.

### Possible biochemical function of BSD2 in vivo

Previous studies have reported that the Rubisco holoenzyme complex contains 8 large subunits and 8 small subunits, and needs multiple chaperone proteins to help it assemble, and one of the important factors is BSD2 [26, 29]. BSD2 has proved to play a critical role in the assembly of eight LS [26]. According to the most recent model, Cpn60αβ/Cpn20 chaperonin firstly act on folding of the newly synthesized LS dimmers. Then RuBisco accumulation factor 1 (Raf1) or ribulose bisphosphate carboxylase factor X (RbcX) helps LS assemble into a higher oligomer. Finally, under the binding of BSD2, the LS_8_ cores are stabilizing into an 8-polymer until the enzyme complex completely assemble. Raf2 may act downstream or upstream of this process [26]. Our findings that BSD2 interacts physically with LS (in BiFC assays) add new evidence to support the notion that preassembly of LS/BSD2 intermediate complex is an early step during Rubisco assembly.

## Conclusions

In short, our combined results suggest that *BSD2* plays an indirect role in the maintenance of BS chloroplast structure by interacting physically with the large submit of Rubisco. Ourdata suggests that *BSD2* is also required for the normal development and intactness of M chloroplasts. Moreover, based on our cytological observation and statistic analysis, we found that *BSD2* most likely plays a role in regulating chloroplast division.

## Methods

### Plant material and growth conditions

Marty Sachs of the Maize Genetics Cooperation Stock Center and Prof. David B. Stern of Boyce Thompson Institute for Plant Research for provided the *bsd2* mutant seeds.

The maize seeds collected from a heterozygous *bsd2* mutant plant (about 25% of the progeny are *bsd2* homozygous mutants) were planted in the Pindstrup Substrate nutritional soil (Denmark) in a growth chamber at 28 °C with 16 h-light (8000Lux)/8 h-dark cycle.

### Light microscope and Electron microscope observation

Leaf sections (2-3 mm^2^) from 6 to 12 days old wild type and *bsd2* mutant plants were fixed in 4% (v/v) glutaraldehyde at 4 °C. The preparation methods of light and electron microscope samples were referred to the previous literature [30]. Sections were examined and photographed using Leica DMLB light microscope and Phillips (Eindhoven, Netherlands) FEI-TECHNAI 12 TEM.

### Expression analysis

Total RNA was isolated from maize leaves using TRIzol reagent (Invitrogen). First-strand cDNA was synthesized from ~ 1 μg of total RNA with an oligo (dT) primer. Real-time PCR was performed on the BioRad CFX384 (USA). *ACTIN* was used as an internal standard for normalizing the cDNA concentrations. The sequences of the gene specific primers are listed in Additional file 1: Table S1. Three biological replicates were used for each sample in the real-time PCR analysis.

### Measurement of chloroplast number and statistic analysis

Leaf sections (2-3 mm^2^) from the middle section of the third leaf in 12 days old wild type and *bsd2* mutant plants were prepared and fixed in FAA (v/v; formaldehyde: glacial acetic acid: 70% ethanol =1:1:18) for 24 h at room temperature. To estimate the number of mature chloroplasts per cell, the samples were separated into single-cell suspensions by treatment with a separation solution (v/v; 10% nitric acid: 10% chromic acid =1:1) at 40 °C for approximately 4-6 h. Next, the samples were washed in water and placed on the microscope slide and stained with Toluidine Blue O. Then, the cover slip was pressed down sufficiently to separate the cells. The number of mature chloroplasts per cell was counted under the eyepiece of a Leica DMLB microscope (Germany).

The number of proplastids per cell was counted under Phillips FEI-TECHNAI 12 TEM (Eindhoven, Netherlands) using ultrathin sections selected from different batches of seedlings. Mean proplastid number was determined based on random selection of 20 cells from 5 sections.

For statistic analysis, each set of measurements represents the average ± SD of 20 cells observed in three different seedlings. Data were analyzed using the SPSS version 10.0 software. The significance level was set at *p* < 0.05.

### BiFC assay

For BiFC assay, the full-length coding sequence of *BSD2* was synthesized and cloned into the BiFC vector pVN to fuse with the N terminus of yellow fluorescent (YFP). The LS coding sequence and an 87 amino acids chloroplast targeting sequence of rice *RBCS* [31, 32] were synthesized and assembled into the BiFC vector pVCto fuse with the C terminus of YFP (GENEWIZ). Both of the vectors were co-transformed into rice protoplasts as described [33]. After incubation of the transformed protoplasts at 30 °C for 24 h, fluorescence images for YFP (green signal) were captured using a laser scanning confocal microscope (LEICA SP8 STED 3X). Auto-fluorescence (red signal) from chloroplast was also obtained.

## Supplementary information

**Additional file 1: Table S1.** The sequences of the gene specific primers.

## Data Availability

The datasets used and/or analysed during the current study available from the corresponding author on reasonable request.

## Abbreviations

ADL: A dynamin-like protein
ARC3: Accumulation and replication of chloroplasts 3
BS: Bundle sheath
*Bsd2*: *Bundle sheath defective2*
FtsZ: Filamenting temperature-sensitive Z
LS: Rubisco large subunit
M: Mesophyll
OAA: Oxaloacetate
PDR1: Plastid-dividing ring 1
PDV: Plastid dividing
PEP: Phosphoenolpyruvate
Rubisco: Ribulose-1, 5-bisphosphate carboxylase/oxygenase
TEM: Transmission electron microscopy
VIPP1: The vesicle-inducing protein in plastid 1
