# Supplementary material for: Cytological evidence of BSD2 functioning in both chloroplast division and dimorphic chloroplast formation in maize leaves
Source: BMC Plant Biol. 2020 Jan 9;20:17. doi: 10.1186/s12870-019-2219-7 (PMC6953307; doi:10.1186/s12870-019-2219-7)
Supplement: Supplementary file 1 — Additional file 1: Table S1. The sequences of the gene specific primers. [file 12870_2019_2219_MOESM1_ESM.pdf]

Table S1. The sequences of the gene specific primers.

Gene	Forward (5'-3')	Reserve (5'-3')
ACTIN	CTCATGCTATTCTCCGTTTGG	TCAGGCATCTCGTAGCTCTTC
PDV1-1	GCAAAGCCGTCTAGTCCTTG	CCTCTGATCGGCTATGGGTA
PDV1-2	TCGGGTGCTTGCTCTCTTAT	CAAGGACTAGACGGCTTTGC
PDV2	TGGGGAACGGTTCTTTGTAG	CAGCACCAAACGCAGAATTA
FtsZ	CGAGTCCATTCAGGAAGCTC	CTTGAACTGCCCGTCTTCTC
ARC3	GGAGGGTGCCTTTGTTCATA	CGTGGAAACTCAAGCACTCA
PDR1	CAGTTGCAGTCAAGCTCTCG	AGCGTCGAACTCAGTCCTGT
ADL1A	TGCAGCTTTTGAGTCATTGG	TTGGGATGGAGTTCCTCAAG
VIPP1	ATAGAGCGAGGCACCAAGAA	TTTGACTGCCGTACTTGCAC
